# Impact of finasteride on modulating the risk and clinical outcomes of bladder cancer: insights from a comprehensive meta-analysis

**DOI:** 10.3389/fphar.2025.1471442

**Published:** 2025-01-29

**Authors:** Ailing Yu, Zian Bai, Yijie Wang, Zhen Luo, Xiaochen Du, Mengxin Chen, Shuang Wen, Honglong Wang, Xiaoying Yuan, Chunyu Yang, Shanshan Bai, Bo Fan

**Affiliations:** ^1^ Department of Urology, Second Affiliated Hospital of Dalian Medical University, Dalian, Liaoning, China; ^2^ Liaoning Provincial Key Laboratory of Urological Digital Precision Diagnosis and Treatment, Dalian, Liaoning, China; ^3^ Liaoning Engineering Research Center of Integrated Precision Diagnosis and Treatment Technology for Urological Cancer, Dalian, Liaoning, China; ^4^ Dalian Key Laboratory of Prostate Cancer Research, Dalian, Liaoning, China; ^5^ First Clinical College, Dalian Medical University, Dalian, Liaoning, China; ^6^ College of Humanities and Social Sciences, Dalian Medical University, Dalian, Liaoning, China; ^7^ Department of Clinical Medicine, First Clinical School of Dalian Medical University, Dalian, Liaoning, China; ^8^ Department of Pathology, Dalian Friendship Hospital, Dalian, China; ^9^ Department of Anatomy, College of Basic Medicine, Dalian Medical University, Dalian, Liaoning, China; ^10^ Bidding and procurement office, Second Affiliated Hospital of Dalian Medical University, Dalian, Liaoning, China; ^11^ Department of Ultrasound, First Affiliated Hospital of Dalian Medical University, Dalian, Liaoning, China

**Keywords:** 5-alpha reductase inhibitors, bladder cancer, incidence, subgroup analysis, meta-analysis

## Abstract

**Purpose:**

Numerous prior analyses have highlighted a potential link between androgen suppression therapy (AST) and bladder cancer (BCa). However, there is a notable gap in research specifically examining the influence of finasteride on BCa risk and clinical outcomes. This study aimed to evaluate preventive and therapeutic value of finasteride for BCa patients.

**Methods:**

This meta-analysis adhered to the Preferred Reporting Items for Systematic Reviews and Meta-Analyses (PRISMA) Guidelines. The PubMed, Embase, Cochrane Library, and Web of Science databases were searched up to 20 December 2024, to identify studies that examined the intake of finasteride and its impact on the incidence and clinical prognosis of patients with BCa. Data was extracted for further analysis by two different reviewers who independently examined the titles and abstracts of the included articles. Subgroup analyses and leave-one-out sensitivity analyses, were applied to mitigate the potential confounding factors associated with heterogeneity.

**Results:**

Our investigation revealed that finasteride markedly decreased the likelihood of developing BCa (hazard ratio [HR]: 0.75, 95% confidence interval [CI]: 0.63–0.88). Subgroup analyses indicated that the preventive effect of finasteride in BCa incidence were generally consistent, regardless of study region, types of research. Furthermore, no notable disparities were observed in OS, CSS, or RFS between the finasteride group and the control group.

**Conclusion:**

Finasteride plays a protective role against the progression of BCa, nevertheless, its effects on prognostic outcomes, including OS, CSS, and RFS, remain inconclusive. Additional multi-center prospective studies with long-term follow-up are required to further validate prophylactic role of finasteride on bladder cancer.

**Systematic review registration number:**

https://www.crd.york.ac.uk/PROSPERO/display_record.php?RecordID=525046, identifier CRD42024525046.

## 1 Introduction

Bladder cancer (BCa) ranked as the fourth most prevalent cancer in men in 2023, accounting for approximately 6% of newly diagnosed cancers and 4% of cancer-related deaths (Lopez-Beltran et al., 2024). Furthermore, bladder cancer was reported to incur the highest expense per patient of all cancers (Mossanen and Gore, 2014). Upon initial diagnosis, between 70% and 75% of BCa patients are found to have non-muscle-invasive bladder cancer (NMIBC), while 20%–25% of patients are diagnosed with muscle-invasive bladder cancer (MIBC), and an additional 5% of patients present with metastatic disease (Lopez-Beltran et al., 2024). Although NMIBC often presents with a more favorable outlook, with many cases managed through chronic monitoring due to a high recurrence rate (Lenis et al., 2020), the situation shifts dramatically with MIBC progression. This transition marks a pivoting toward a more aggressive disease spectrum, wherein the 5-year survival rates decline to approximately 50% (Wissing et al., 2019), thus necessitating multimodal and invasive treatment strategies. Given the grim outlook associated with MIBC, treatment strategies encompass a comprehensive approach that includes radical surgery, radiotherapy, and chemotherapy. Despite these measures, approximately half of the affected individuals experience metastasis and succumb to the disease within 3 years (Fan et al., 2021). Consequently, the implementation of early detection techniques and continuous monitoring of patient prognosis are crucial for decreasing the mortality rates associated with bladder cancer (BCa).

Numerous *in vitro* and *in vivo* studies have underscored the significance of androgen receptors (ARs) in the onset, progression, and relapse of BCa, as well as its resilience against standard treatments, including radiotherapy, chemotherapy, and *Bacillus* Calmette-Guerin therapy (Kourbanhoussen et al., 2021; Ide and Miyamoto, 2021; Costa et al., 2020; Koti et al., 2020; Luna-Velez et al., 2021; Deng et al., 2021; Ide et al., 2018; Tripathi and Gupta, 2020). 5-Alpha reductase inhibitors (5-ARIs) are used as androgen suppression therapy (AST) that targets and effectively impedes the production of dihydrotestosterone (DHT), the foremost endogenous activator of androgen receptors (ARs). Recent clinical studies indicated that attenuating androgenic signaling (Morales et al., 2016) could impact the behavior of BCa. However, whether 5-ARIs affect the prognosis and risk of BCa is still unclear (Sathianathen et al., 2018; Wissing et al., 2019; Wissing et al., 2021; Wang et al., 2020; Dekalo et al., 2023). Evidence from preclinical research indicated that the efficacy of antiandrogen drugs against BCa varies (Imada et al., 1997). Based on population data, Dekalo’s retrospective cohort study explored the associations between the use of 5ARIs, BCa occurrence, and related mortality. The study found that the intake of finasteride but not dutasteride was related to a decrease in BCa risk (Dekalo et al., 2023). The findings suggest that a significant variation in outcomes observed across prior studies can be attributed to the simultaneous examination of 5-ARIs and androgen deprivation therapy (ADT), as well as the effects of finasteride and dutasteride. Numerous prior analyses have highlighted a potential link between AST and BCa (Kim et al., 2020; Creta et al., 2021; Xiang et al., 2021). However, there is a notable gap in research specifically examining the influence of finasteride on BCa risk and prognosis. Thus, as new research emerges, an updated synthesis of the findings is needed.

The purpose of our study was to synthesize existing data and deliver an in-depth systematic review of the effects of finasteride on the occurrence of BCa and patient prognosis. This research findings should significantly enhance our comprehension of the risk and prognostic factors of BCa and present strategies for its prevention or treatment.

## 2 Materials and methods

### 2.1 Search strategy

This meta-analysis was conducted according to the Preferred Reporting Items for Systematic Reviews and Meta-Analyses (PRISMA) guidelines and registered in the PROSPERO database under CRD42024525046. Searches of the PubMed, Embase, Web of Science, and Cochrane Library databases were performed until 20 December 2024, aiming to locate studies that evaluated the impact of finasteride on the incidence and prognosis of patients with BCa. On PubMed, the following search terms were used: (Urinary Bladder Neoplasms OR Neoplasm, Urinary Bladder OR Urinary Bladder Neoplasm OR Bladder Tumors OR Bladder Tumor OR Tumor, Bladder OR Tumors, Bladder OR Neoplasms, Bladder OR Bladder Neoplasms OR Bladder Neoplasm OR Neoplasm, Bladder OR Urinary Bladder Cancer OR Cancer, Urinary Bladder OR Malignant Tumor of Urinary Bladder OR Cancer of the Bladder OR Bladder Cancer OR Bladder Cancers OR Cancer, Bladder OR Cancer of Bladder) AND (Finasteride OR Chibro Proscar OR Propecia OR MK-906 OR MK 906 OR MK906 OR Proscar) AND (randomized controlled trial OR randomized OR placebo). Following the removal of duplicate data, two independent reviewers, unbound by national or linguistic limitations, scrutinized the titles and abstracts. They dismissed studies unrelated to our research, reviews, and animal-related experiments to enhance data quality.

### 2.2 Criteria for inclusion and exclusion

The inclusion criteria includes: 1) Population: patients diagnosed with bladder tumors as urothelial carcinoma through histological or pathological assessments; 2) Intervention group setup: administration of finasteride; 3) Outcomes: availability of sufficient data for computing odds ratios (ORs)/relative risks (RRs)/hazard ratios (HRs), along with 95% confidence intervals (CIs) and P values, with median follow-up period of ≥1 year and sample size of ≥100; 4) Type of research: randomized control trial or case-control study or cohort study. The exclusion criteria were as follows: 1) publications such as review articles, editor’s letters, commentaries, or case studies lacking original data; 2) studies focusing on molecular biology, particularly those examining finasteride’s effects on cancerous cell lines or in animal models; 3) the HR/OR/RR and its standard error could not be obtainable from the provided details.

### 2.3 Data extraction and quality assessment

**FIGURE 1:**
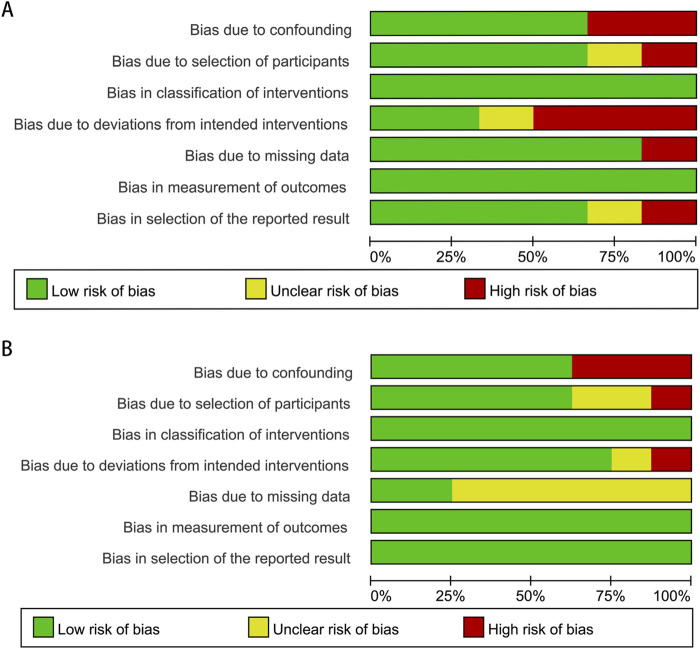
Methodological quality graph for the meta-analysis of the incidence **(A)** and prognosis **(B)** of bladder cancer.

Following a detailed assessment of the full texts, both authors independently extracted the data for subsequent evaluation to enhance the reliability of the data. From each chosen article, we gathered information on various aspects, including the first author, study region, types of research, study period, average age of patients, sample size, median follow-up period, dose of finasteride and adjusted HR/RR/OR (95% CI). To evaluate the methodological integrity of these studies, we employed the risk of bias in nonrandomized studies of interventions (ROBINS-I) assessment tool. The findings have been compiled and are presented in Figure 1.

### 2.4 Statistical analysis

In our analysis, we categorized bladder cancer (BCa) occurrences into two groups: those associated with finasteride usage and those without. To depict the incidence and intensity of BCa in both the finasteride-utilizing and non-utilizing cohorts, we constructed forest plots, employing hazard ratios (HRs) and 95% confidence intervals (CIs) as our metrics. We measured heterogeneity using the I^2^ statistic. In instances of notable heterogeneity, a random effects model was employed; for other situations, a fixed effects approach was implemented. To ensure the precision of our findings and account for potential confounding effects from the inclusion of studies evaluating drugs beyond finasteride, such as dutasteride, we performed a leave-one-out sensitivity analysis, which evaluated the impact of each specific study on the total heterogeneity. This dual approach allowed us to evaluate the robustness of our conclusions while minimizing potential biases. To investigate potential publication bias, Begg’s and Egger’s tests were conducted when the number of included studies was above 10. Stata 14.0 software was utilized to conduct these analyses.

## 3 Results

### 3.1 Study identification and selection

**FIGURE 2:**
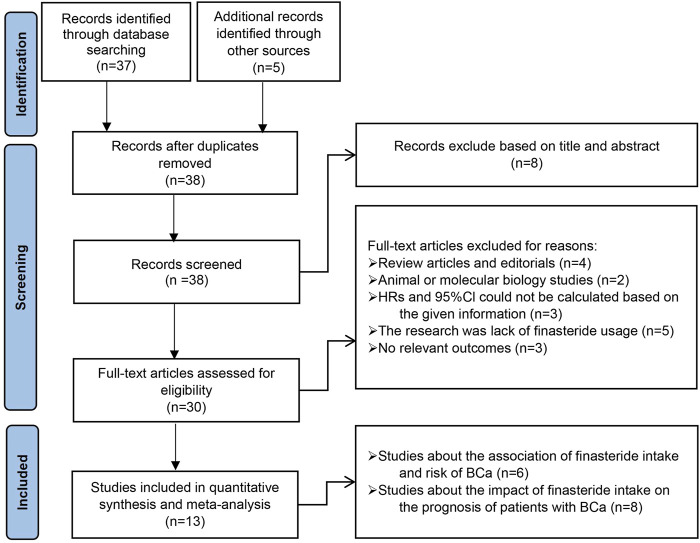
Preferred Reporting Items for Systematic Review and Meta-Analysis (PRISMA) flow diagram for the selection of articles.

The PRISMA study selection process is outlined in Figure 2. Our initial search of the databases yielded 37 relevant studies. Five records identified from alternative sources are also included. Following the examination of titles, abstracts, and complete texts, duplicate studies and unrelated articles (such as commentaries, reviews, animal or cellular studies) that did not fit our research scope were excluded. Ultimately, our comprehensive analysis included six studies examining the link between finasteride use and the risk of BCa and eight studies exploring the influence of finasteride treatment on the prognosis of patients with BCa. All of the included studies met the predefined eligibility criteria.

### 3.2 Study characteristics

Table 1 details the features of the six studies focused on exploring the link between finasteride consumption and the risk of BCa. These studies were published from 2016 to 2023. Four of the studies were performed in the United States, one was conducted in Canada, and one was conducted in Taiwan. Among the studies, all but one were retrospective cohort studies, with the remaining one being a case-control study. The included studies had sample sizes varying between 2,700 and 186,394. Table 2 shows the characteristics of the eight studies that assessed the effect of finasteride on BCa patient outcomes. These studies were published predominantly between 2018 and 2023 and were performed in Canada (four studies), the United States (two), Finland (one), and Taiwan (one). The sample sizes varied between 206 and 186,394, and all of the studies were retrospective cohort studies.

**TABLE 1:** Main characteristics of individual studies included in the meta-analysis on impact of finasteride intake on risk of BCa.

First author (year)	Study region	Types of research	Study period	Average age of patients (year)	Sample size	Median follow-up period (year)	Dose of finasteride (daily)	Adjusted HR/RR/OR (95% CI)
Dekalo S 2023	Canada	Retrospective cohort	2003–2020	75	186,394	1.7	Finasteride:5 mg	0.88 (0.81–0.95)
Zhu D 2021	America	Retrospective cohort	2000–2016	67	42,406	5.4	Finasteride:NA	0.64 (0.51–0.80)
Sathianathen NJ 2018	America	Retrospective cohort	1993–1998	62.6	2,700	6	Finasteride: 5 mg	1.22 (0.48–3.09)
Chen CC 2018	Taiwan	Case-control study	2002–2013	68.6	33,568	6	Finasteride: NA	0.84 (0.70–0.99)
Srivastava A 2017	America	Retrospective cohort	2000–2016	NA	42,774	7.3	Finasteride:NA	0.57 (0.45–0.71)
Morales EE 2016	America	Retrospective cohort	1993–2006	63/62	72,370	13	Finasteride:NA	0.73 (0.55–0.97)

NA, not available; HR, hazard ratio; RR, relative risk; OR, odds ratio.

**TABLE 2:** Main characteristics of eligible studies collected in the meta-analysis on effect of finasteride intake on prognosis of BCa.

First author (year)	Study region	Types of research	Study period	Average age of patients (year)	Sample size	Median follow-up period (year)	Dose of finasteride	Adjusted HR/RR/OR (95% CI)
Dekalo S 2023	Canada	Retrospective cohort	2003–2020	75	186,394	3/6	Finasteride:5 mg	CSS:0.82 (0.65–1.02)
Garg H 2023	America	Retrospective cohort	2004–2015	74	1890	4.5	Finasteride:NA	OS: 0.74 (0.63–0.86)
Wissing MD 2021	Canada	Retrospective cohort	2000–2015	70	2,822	7.7	Finasteride:NA	OS: 1.03 (0.88–1.21) CSS: 1.12 (0.92–1.36) RFS: 1.19 (0.99–1.42)
Wu SC 2021	America	Retrospective cohort	2001–2017	68.3	274	3.1	Finasteride:NA	RFS: 0.53 (0.30–0.88)
Wang CS 2020	Taiwan	Retrospective cohort	1998–2010	76.5/76.6	5214	3	Finasteride:5 mg	CSS: 0.84 (0.71–0.98) RFS: 0.96 (0.82–1.11)
Al-Hogbani M 2020	Canada	Retrospective cohort	2013–2018	70	206	3.3	Finasteride:5 mg	RFS: 1.00 (0.55–1.79)
McMartin C 2019	Canada	Retrospective cohort	2009–2017	72.5/68.7	338	1.8	finasteride: 5 mg	OS: 0.40 (0.19–0.83)
Mäkelä VJ 2018	Finland	Retrospective cohort	1997–2012	75/70	10,720	4.2	Finasteride:5 mg	CSS: 0.78 (0.68–0.89)

NA, not available; OS, overall survival; CSS, cancer-specific survival; RFS, recurrence-free survival.

### 3.3 The link between finasteride and bladder cancer risk

#### 3.3.1 Overall assessment

**FIGURE 3:**
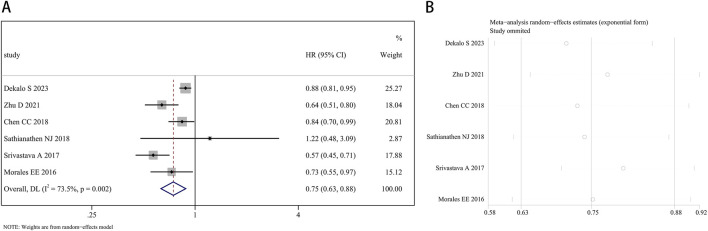
Forest plot **(A)** and leave-one-out sensitivity analysis **(B)** showing the relationship between finasteride intake and the risk of bladder cancer in the overall analysis.

Six studies reported a link between finasteride and the occurrence of BCa. The random effects approach was utilized, as there was significant heterogeneity (I^2^ = 73.5%, P = 0.002). Overall, the combined HR was 0.75 (95% CI: 0.63–0.88, P = 0.001; Figure 3A), indicating an association between finasteride treatment and a 25% reduction in the likelihood of BCa development. The heterogeneity of each study’s results was evaluated using a leave-one-out sensitivity analysis, as depicted in Figure 3B.

#### 3.3.2 Subgroup analysis

**FIGURE 4:**
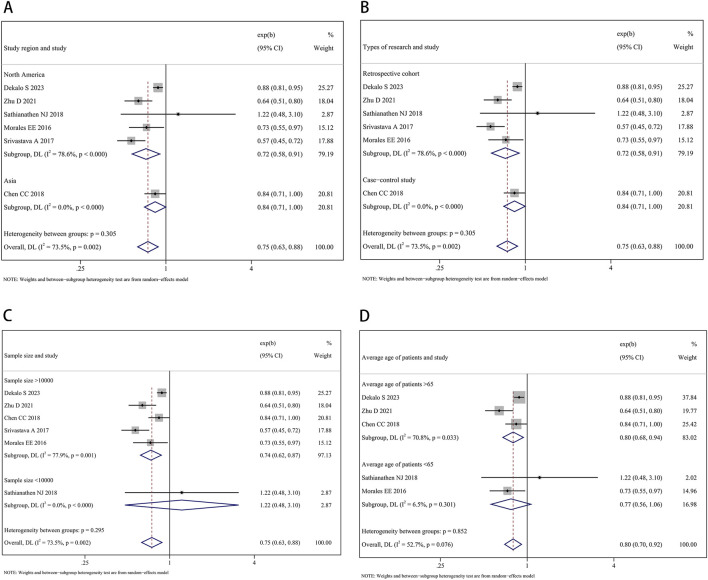
Forest plots showing the impact of finasteride on the occurrence of bladder cancer by subgroup analysis of the **(A)** study region, **(B)** types of research, **(C)** sample size, and **(D)** average age of patients.

The subgroup analysis by study region found a significant impact of finasteride on the decreased occurrence of BCa both in North America (HR: 0.72, 95% CI: 0.58–0.91, P = 0.005) and Asia (HR: 0.84, 95% CI: 0.71–1.00, P = 0.049). In the analysis of types of research, precise evidence was obtained showing that finasteride treatment was related to reductions in BCa risk in both the subgroup of retrospective cohort studies (HR: 0.72, 95% CI: 0.58–0.91, P = 0.005) and the case-control study (HR: 0.84, 95% CI: 0.71–1.00, P = 0.049). In the subgroup analysis by sample size, when the sample size was more than 10,000, the risk of BCa was 0.74 times in patients with finasteride intake compared to those without (95% CI: 0.62–0.87, P < 0.001). When the sample size was 10,000 or less, there is no significant statistical difference in BCa risk (HR: 1.22, 95% CI: 0.48–3.10, P = 0.676). In the subgroup analysis by average age of patients, a significantly reduced risk of BCa was found in patients aged >65 years old who took finasteride (HR: 0.80, 95% CI: 0.68–0.94, P = 0.008). However, no significant difference in risk was found in patients aged <65 years old (HR: 0.77, 95% CI: 0.56–1.06, P = 1.112). Figures 4A–D show the forest plots.

### 3.4 The effect of finasteride on the outcomes of patients with bladder cancer

#### 3.4.1 Finasteride and the survival of bladder cancer patients

**FIGURE 5:**
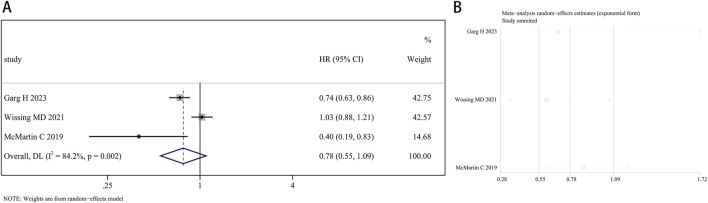
Forest plots for the relationship between finasteride intake and overall survival in patients with bladder cancer. **(A)** Meta-analysis. **(B)** Sensitivity analysis.

**FIGURE 6:**
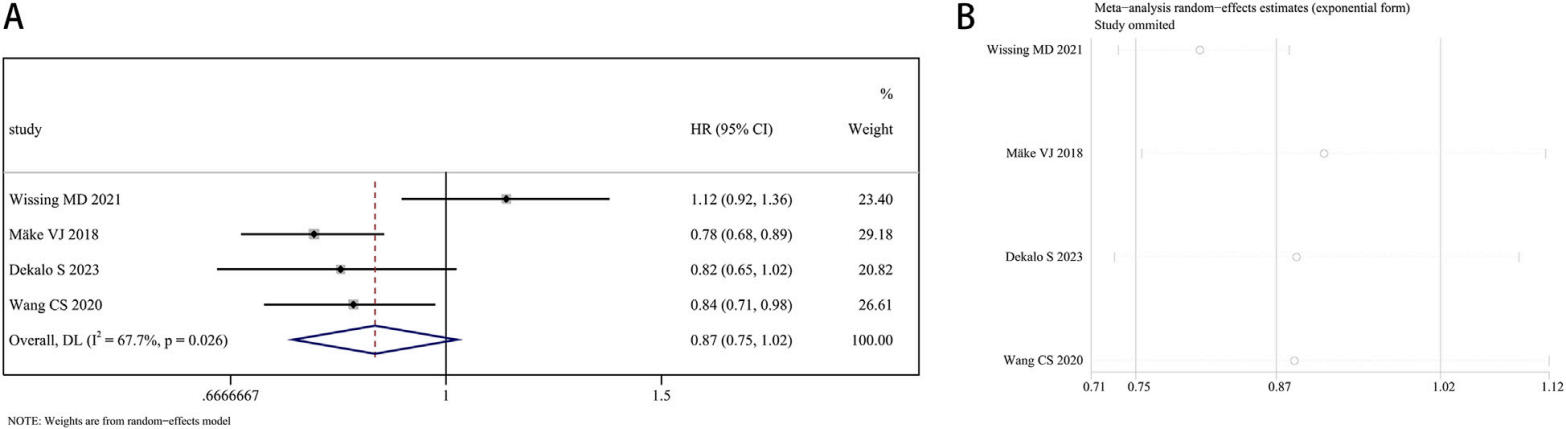
Forest plots for the association between finasteride intake and cancer-specific survival in patients with bladder cancer. **(A)** Meta-analysis. **(B)** Sensitivity analysis.

A total of 207,858 participants with survival information were included in eight articles. Three of these studies examined the connection between finasteride consumption and overall survival (OS) in BCa patients, while four studies provided data on cancer-specific survival (CSS). A random effects model was employed for OS analysis due to notable heterogeneity (I^2^ = 84.2%, P = 0.002). The combined data revealed no marked difference in OS between patients treated with finasteride and the control group (HR: 0.78, 95% CI: 0.55–1.09, P = 0.149; Figure 5A). A leave-one-out sensitivity analysis was utilized to assess the heterogeneity effect in each study (Figure 5B). Additionally, treatment with finasteride did not notably affect CSS in patients (HR: 0.87, 95% CI: 0.75–1.02, P = 0.089; Figure 6A). Significant heterogeneity was also noted in CSS (I^2^ = 67.7%, P = 0.026). Each study’s heterogeneity was scrutinized through a leave-one-out sensitivity analysis. The outcomes are shown in Figure 6B.

#### 3.4.2 Finasteride and the recurrence-free survival of bladder cancer patients

**FIGURE 7:**
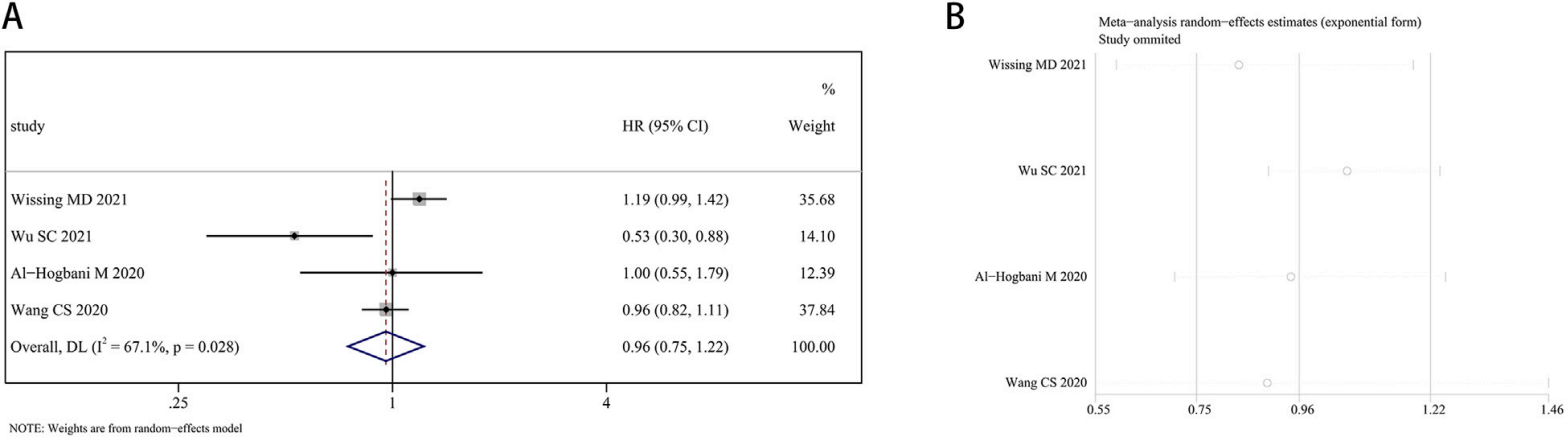
Forest plots for the association between finasteride intake and recurrence-free survival in patients with bladder cancer. **(A)** Meta-analysis. **(B)** Sensitivity analysis.

Four research articles presented the estimated impacts of finasteride on bladder cancer recurrence. Given the significant heterogeneity (I^2^ = 67.1%, P = 0.028), the random effects approach was utilized. Finasteride did not significantly increase recurrence-free survival (RFS) in BCa patients (HR: 0.96, 95% CI: 0.75–1.22; P = 0.731) compared to those who did not take finasteride (Figure 7A). Each study’s heterogeneity was evaluated using a leave-one-out sensitivity analysis, which is detailed in Figure 7B.

## 4 Discussion

The investigation of 5-ARI, as crucial component of ASTs utilized in the clinical management of benign prostatic hyperplasia (BPH) (Kim et al., 2016), has recently shifted toward investigating the potential of 5-ARIs in a new therapeutic context—addressing the incidence of BCa. However, the conclusions are inconsistent (Zhu et al., 2021; Dekalo et al., 2023; Sathianathen et al., 2018). We conducted a meta-analysis incorporating data from several studies to elucidate the specific influence of finasteride treatment on BCa outcomes. Specifically, individuals with a history of finasteride use exhibited an HR of 0.75 (P = 0.001) compared to nonusers, suggesting a 25% reduction in BCa risk.

Some potential mechanisms may explain our findings. 1) The impact on the AR signaling pathway. As a member of the 5-ARI class of drugs, finasteride could influence AR signaling, which had been implicated in several key genomic events, including the induction of DNA breaks, alterations in chromosomal structure, and the inhibition of uridine 5′-diphospho-glucuronosyltransferase activity, a pivotal enzyme in the detoxification process (Lin et al., 2009; Izumi et al., 2013). These findings suggest that AR signaling may contribute to carcinogenesis by compromising genomic stability and impairing the cellular capacity to detoxify carcinogens. Meanwhile, analysis of The Cancer Genome Atlas (TCGA) data revealed modifications in 5-AR genes at both the DNA and RNA levels, characterized by gene amplification and elevated mRNA expression, which underscores the genetic basis for targeting AR signaling in BCa (Chen et al., 2017). This dual effect of 5-ARIs offers a promising avenue for BCa management, suggesting a direct impact on AR signaling as a mechanism of action. 2) Interference with the tumor microenvironment. As BCa is a heterogeneous malignancy characterized by distinct immune subtypes and differences in the tumor immune microenvironment landscape, various components, including tumor cells, stromal cells, immune cells, and the extracellular matrix, have prognostic surveillance. A previous study indicated that finasteride could potentially inhibit the expression of Insulin-like Growth Factor 1(IGF-1) by suppressing the activity of c-JUN within fibroblasts in benign prostate tissue, leading to a reduction in the synthesis and secretion of IGF-1 (Wang et al., 2017). IGF-1 has been found to promote the proliferation of prostate cancer cells and inhibit apoptosis (Wissmiller et al., 2023). Another study found that finasteride reduced the secretion of CCL5 by CD8^+^ T cells by upregulating the cGMP/PKG/p6 5 signaling pathway (Jin et al., 2019). CCL5 is believed to promote the proliferation and metastasis of bladder cancer through the JAK2/STAT3 signaling pathway (Shen et al., 2023), which might suggest the reliability of finasteride in reducing the occurrence of BCa from the perspective of the immune microenvironment. 3)Produce crosstalk between tumors and microorganisms. In contemporary scientific discourse, inflammation is regarded as a major contributing factor to carcinogenesis, with the microorganism being recognized as an integral element in this process (Elinav et al., 2013). Recent research findings indicated that finasteride may possess anti-inflammatory properties that reduce multiple inflammatory factors, including NOS2, NOX4, and COX2, in murine models of BPH (Lee et al., 2023). A separate study observed that the cessation of finasteride intake could result in the onset of intestinal inflammation in adult male mice, which was characterized by elevated expression levels of interleukin-6β, tumor necrosis factor-α, and other pro-inflammatory cytokines (Diviccaro S et al., 2022). Furthermore, microbial imbalance within the bladder could contribute to the development of BCa. Nardelli and colleagues examined the relationship between the urinary microbiome and bladder cancer (Nardelli et al., 2024). They found an increased abundance of bacteria from the Porphyromonas genus, particularly *Porphyromonas somerae*, in urine from BCa patients. Of particular interest is the association of *P. somerae* with the progression of endometrial cancer, where it has been implicated in the promotion of carcinogenesis through mechanisms including the induction of inflammation, the modulation of host cell functions, and the enhancement of oxidative stress. Additionally, droplet digital Polymerase Chain Reaction and next-generation sequencing methodologies showed that the presence and abundance of *P. somerae* in urine samples from BCa patients were significantly higher than in urine samples from the control group (Russo et al., 2024), which support that Porphyromonas somerae has the potential to serve as a distinctive microbiome marker for BCa. 4)The interaction between androgens, estrogens, and the immune system. 5-ARIs have been shown to increase estrogen levels in the serum of males, which, combined with significant changes in local sex steroid concentrations, may enhance local immune responses against tumor cells (Shibata et al., 2017; Kristal et al., 2012). The incidence of bladder cancer exhibits a gender correlation—with a higher incidence rate in males. In males, the loss of the Y chromosome (LOY) occurs at a high rate of up to 40% (Minner et al., 2010). A study indicates that the LOY can induce terminal exhaustion of CD8^+^ T cells, leading to immune evasion in bladder cancer. Concurrently, the loss of the Y chromosome increases the sensitivity of bladder cancer patients to anti-PD1 immune checkpoint inhibitor therapy (Abdel-Hafiz et al., 2023). There are articles explaining that, given urothelial carcinoma’s response to checkpoint inhibitors, the modulation of sex hormone levels by 5-ARIs could synergize with immunotherapeutic strategies to counteract tumor-induced immune suppression (Bellmunt et al., 2017).

We conducted several subgroup and leave-one-out sensitivity analyses to mitigate the potential confounding factors associated with heterogeneity. First, the subgroup analysis by study region indicated the significant impact of finasteride on the decreased occurrence of BCa in patients in both North America and Asia, which indicated that ethnicity could be a significant factor influencing the BCa risk influence of finasteride. This emphasizes the importance of considering ethnic diversity in assessing the efficacy and safety profile of the drug. Zhu proposed that polymorphisms within the AR gene, which activates ARs independent of DHT binding, may contribute to the observed variance (Zhu et al., 2021). This insight underscores a potential genetic mechanism that could influence the response to therapies targeting the androgen pathway. Further research is warranted to explore the mechanisms behind these variations and to determine whether specific ethnic populations may benefit more or face greater risks from finasteride therapy. Second, subgroup analysis by type of research, average age of patients, and sample size was conducted to verify their impact on the study findings. The results indicated that finasteride treatment reduced the incidence of BCa by 28% in a retrospective cohort subgroup, by 16% in a case-control subgroup, by 20% in a subgroup >65 years old, and by 26% in sample size >10,000 subgroup. Meanwhile, we adopted a leave-one-out sensitivity analysis to mitigate this effect and assess the solidity of our conclusions, reinforcing the robustness of our study.

An increasing number of studies have focused on the impact of finasteride on the prognosis of patients with BCa. Garg’s research indicated a positive correlation between the use of finasteride and enhanced OS among patients diagnosed with NMIBC (Garg et al., 2023). Another observation by McMartin et al. (McMartin et al., 2019) suggested that 5-ARIs might also confer beneficial biological effects on individuals with MIBC. Contrary to previous meta-analysis findings (Creta et al., 2021) that indicated a lower risk of BCa recurrence with 5-ARI treatment, our study found that the finasteride treatment did not significantly improve OS, CSS, or RFS, which collectively indicated that finasteride did not appear to affect survival outcomes or the likelihood of cancer recurrence. Possible explanations for these findings include differences in the follow-up periods in the included studies, particularly the restricted number of cases with a follow-up duration exceeding 10 years. Concurrently, the limited number of studies restricted us from stratifying risk factors related to BCa, such as T stage, pathological grading, lymph node invasion, or distant metastasis. We also refined the inclusion criteria by omitting two studies due to their lack of finasteride usage, aiming to enhance the accuracy of our conclusions. The differences in findings between our analysis and prior studies may stem from these methodological adjustments and the inclusion of new data, highlighting the evolving nature of medical evidence.

Several study limitations should be considered. First, although we did not restrict the language of the included studies and expanded our literature search to unpublished trials and abstracts, potential bias could not be avoided. Second, although we performed subgroup and sensitivity analyses to try to control for potential confounding factors, the efficacy of mitigating confounding factors that may interfere with the application of research findings was limited by the number of included studies. Third, the absence of detailed information on tumor characteristics, including pathological grade or T stage, limited our understanding of the impact of finasteride on BCa in our pooled analysis. Fourth, the heterogeneity among the included samples, possibly due to the varied inclusion of 5-ARIs, may have obscured the true association between finasteride use and BCa outcomes. Lastly, factors such as the dose of finasteride used and the duration of administration were not be done by subgroups analysis due to the limited number of samples included. Chen et al. (2017) categorized 5-alpha-reductase inhibitor (5ARI) usage into three tiers by cumulative defined daily doses (cDDD) as 0, 1 to 179, and over 180 which is calculated as the sum of dispensed DDD over time, with DDD being the assumed average daily maintenance dose of a drug used for its main indication in adults, and reported that patients with daily doses >180 had a lower propensity for BCa development. Meanwhile, Snir Dekalo’s study (Dekalo et al., 2023) revealed a significant protective effect of 5ARIs after a minimum of 2 years of exposure. These findings emphasize the need for further research to assess the long-term effects of continuous finasteride therapy or dose-response relationship of 5ARI with BCa risk.

The findings of this study, which indicate a significant effect of finasteride in preventing the occurrence of BCa, as well as high incidence and mortality rates, support the feasible of finasteride chemoprevention trials in populations at high risk for bladder cancer. The specific mechanisms of finasteride in preventing BCa are currently unclear; hence, further molecular biology experiments should be performed. A large-sample, multicenter, prospective study of finasteride uptake should be conducted. Due to the lack of detailed data on cancer grading, staging, or histology in existing studies, there is an expectation that future studies will provide more detailed clinical reports to assist researchers in further investigating bladder cancer. Regular long-term follow-up and detailed subgroup stratification will help to assess the true value of finasteride in preventing the development of BCa and provide new ideas for future integrated treatment, including postoperative bladder perfusion therapy for NMIBC, preoperative neoadjuvant chemotherapy for MIBC, and adjuvant chemotherapy for advanced metastatic BCa.

## 5 Conclusion

Our comprehensive review and meta-analysis included 13 studies that evaluated the impact of finasteride on the risk and clinical outcomes of patients with BCa. The analysis indicated that finasteride contributes positively to reducing the incidence of BCa. Our subgroup analysis results suggested that finasteride shows a significant potential for preventing BCa across different regions. No notable differences were observed in OS, CSS, or RFS between the finasteride group and controls. Future research should include rigorously designed prospective studies that account for prevalent confounding variables to corroborate these findings.

## Data Availability

The original contributions presented in the study are included in the article/supplementary material, further inquiries can be directed to the corresponding authors.

## References

[B1] Abdel-Hafiz H. A. Schafer J. M. Chen X. Xiao T. Gauntner T. D. Li Z. (2023). Y chromosome loss in cancer drives growth by evasion of adaptive immunity. 619, 624–631. 10.1038/s41586-023-06234-x PMC10975863 37344596

[B2] Bellmunt J. de Wit R. Vaughn D. J. Fradet Y. Lee J.-L. Fong L. (2017). Pembrolizumab as second-line therapy for advanced urothelial carcinoma. N. Engl. J. Med. 376, 1015–1026. 10.1056/NEJMoa1613683 28212060 PMC5635424

[B3] Chen C.-C. Huang C.-P. Tsai Y.-T. Hseih T.-F. Shyr C.-R. (2017). The genomic alterations of 5α-reductases and their inhibitor finasteride's effect in bladder cancer. Cancer. Anticancer Res. 37, 6893–6898. 10.21873/anticanres.12152 29187470

[B4] Costa A. R. Lança de Oliveira M. Cruz I. Gonçalves I. Cascalheira J. F. Santos C. R. A. (2020). The sex bias of cancer. Trends Endocrinol. Metab. Tem. 31, 785–799. 10.1016/j.tem.2020.07.002 32900596

[B5] Creta M. Celentano G. Napolitano L. La Rocca R. Capece M. Califano G. (2021). Inhibition of androgen signalling improves the outcomes of therapies for bladder cancer: results from a systematic review of preclinical and clinical evidence and meta-analysis of clinical studies. Diagn. Basel Switz. 11, 351. 10.3390/diagnostics11020351 PMC7923424 33672461

[B6] Dekalo S. McArthur E. Campbell J. Ordon M. Power N. Welk B. (2023). 5α-reductase inhibitors and the risk of bladder cancer in a large, population-based cohort. Urol. Oncol. Semin. Orig. Investig. 41, 50.e11–50.e17. 10.1016/j.urolonc.2022.09.004 36319553

[B7] Deng G. Wang R. Sun Y. Huang C.-P. Yeh S. You B. (2021). Targeting androgen receptor (AR) with antiandrogen Enzalutamide increases prostate cancer cell invasion yet decreases bladder cancer cell invasion via differentially altering the AR/circRNA-ARC1/miR-125b-2-3p or miR-4736/PPARγ/MMP-9 signals. Cell Death Differ. 28, 2145–2159. 10.1038/s41418-021-00743-w 34127806 PMC8257744

[B8] Diviccaro S. Giatti S. Cioffi L. Falvo E. Herian M. Caruso D. (2022). Gut inflammation induced by finasteride withdrawal: therapeutic effect of allopregnanolone in adult male rats. Biomolecules 12, 1567. 10.3390/biom12111567 36358917 PMC9687671

[B9] Elinav E. Nowarski R. Thaiss C. A. Hu B. Jin C. Flavell R. A. (2013). Inflammation-induced cancer: crosstalk between tumours, immune cells and microorganisms. Nat. Rev. Cancer 13, 759–771. 10.1038/nrc3611 24154716

[B10] Fan B. Mohammed A. Huang Y. Luo H. Zhang H. Tao S. (2021). Can aspirin use Be associated with the risk or prognosis of bladder cancer? A case-control study and meta-analytic assessment. Front. Oncol. 11, 633462. 10.3389/fonc.2021.633462 34350107 PMC8327774

[B11] Garg H. Wheeler K. M. Dursun F. Cooper R. E. Pruthi D. K. Kaushik D. (2023). Impact of finasteride on survival in bladder cancer: a retrospective multi-institutional database analysis. Clin. Genitourin. Cancer 21, 314.e1–314.e7. 10.1016/j.clgc.2022.10.014 36402643

[B12] Ide H. Inoue S. Mizushima T. Jiang G. Chuang K. H. Oya M. (2018). Androgen receptor signaling reduces radiosensitivity in bladder cancer. Mol. Cancer Ther. 17, 1566–1574. 10.1158/1535-7163.MCT-17-1061 29720561

[B13] Ide H. Miyamoto H. (2021). Sex hormone receptor signaling in bladder cancer: a potential target for enhancing the efficacy of conventional non-surgical therapy. Cells 10, 1169. 10.3390/cells10051169 34064926 PMC8150801

[B14] Imada S. Akaza H. Ami Y. Koiso K. Ideyama Y. Takenaka T. (1997). Promoting effects and mechanismsof action of androgen in BladderCarcinogenesis in male rats. Eur. Urol. 31, 360–364. 10.1159/000474484 9129932

[B15] Izumi K. Zheng Y. Hsu J.-W. Chang C. Miyamoto H. (2013). Androgen receptor signals regulate UDP-glucuronosyltransferases in the urinary bladder: a potential mechanism of androgen-induced bladder carcinogenesis. Mol. Carcinog. 52, 94–102. 10.1002/mc.21833 22086872

[B16] Jin S. Xiang P. Liu J. Yang Y. Hu S. Sheng J. (2019). Activation of cGMP/PKG/p65 signaling associated with PDE5‐Is downregulates CCL5 secretion by CD8+ T cells in benign prostatic hyperplasia. Prostate 79, 909–919. 10.1002/pros.23801 30958912 PMC6593656

[B17] Kim A. Kim M. S. Ahn J.-H. Choi W. S. Park H. K. Kim H. G. (2020). Clinical significance of 5-α reductase inhibitor and androgen deprivation therapy in bladder cancer incidence, recurrence, and survival: a meta-analysis and systemic review. Aging Male Off. J. Int. Soc. Study Aging Male 23, 971–978. 10.1080/13685538.2019.1646238 31724468

[B18] Kim E. H. Larson J. A. Andriole G. L. (2016). Management of benign prostatic hyperplasia. Annu. Rev. Med. 67, 137–151. 10.1146/annurev-med-063014-123902 26331999

[B19] Koti M. Ingersoll M. A. Gupta S. Lam C. M. Li X. Kamat A. M. (2020). Sex differences in bladder cancer immunobiology and outcomes: a collaborative review with implications for treatment. Eur. Urol. Oncol. 3, 622–630. 10.1016/j.euo.2020.08.013 32967818

[B20] Kourbanhoussen K. McMartin C. Lodde M. Zlotta A. Bryan R. T. Toren P. (2021). Switching cancers: a systematic review assessing the role of androgen suppressive therapy in bladder cancer. Eur. Urol. Focus 7, 1044–1051. 10.1016/j.euf.2020.10.002 33132108

[B21] Kristal A. R. Till C. Tangen C. M. Goodman P. J. Neuhouser M. L. Stanczyk F. Z. (2012). Associations of serum sex steroid hormone and 5a- androstane-3a,17b-diol glucuronide concentrations with prostate cancer risk among men treated with finasteride. Cancer Epidemiol. 10.1158/1055-9965.EPI-12-0695 PMC3467348 22879203

[B22] Lee G.-H. Lee H.-Y. Zhao L. Rashid M. M. U. Kim M. K. Jeong Y. B. (2023). The role of reactive oxygen species, inflammation, and endoplasmic reticulum stress response in the finasteride protective effect against benign prostate hyperplasia. Eur. Urol. Open Sci., 18–26. 10.1016/j.euros.2023.11.003 PMC11216955 37853537

[B23] Lenis A. T. Lec P. M. Chamie K. Mshs M. D. (2020). Bladder cancer: a review. JAMA 324, 1980–1991. 10.1001/jama.2020.17598 33201207

[B24] Lin C. Yang L. Tanasa B. Hutt K. Ju B. Ohgi K. (2009). Nuclear receptor-induced chromosomal proximity and DNA breaks underlie specific translocations in cancer. Cell 139, 1069–1083. 10.1016/j.cell.2009.11.030 19962179 PMC2812435

[B25] Lopez-Beltran A. Cookson M. S. Guercio B. J. Cheng L. (2024). Advances in diagnosis and treatment of bladder cancer. BMJ 384, e076743. 10.1136/bmj-2023-076743 38346808

[B26] Luna-Velez M. V. Dijkstra J. J. Heuschkel M. A. Smit F. P. van de Zande G. Smeets D. (2021). Androgen receptor signalling confers clonogenic and migratory advantages in urothelial cell carcinoma of the bladder. Mol. Oncol. 15, 1882–1900. 10.1002/1878-0261.12957 33797847 PMC8253097

[B27] McMartin C. Lacombe L. Fradet V. Fradet Y. Lodde M. Toren P. (2019). Receipt of 5-alpha reductase inhibitors before radical cystectomy: do they render high-grade bladder tumors less aggressive? Clin. Genitourin. Cancer 17, e1122–e1128. 10.1016/j.clgc.2019.07.016 31594737

[B28] Minner S. Kilgué A. Stahl P. Weikert S. Rink M. Dahlem R. (2010). Y chromosome loss is a frequent early event in urothelial bladder cancer, , 42, 356. 10.3109/00313021003767298 20438408

[B29] Morales E. E. Grill S. Svatek R. S. Kaushik D. Thompson I. M. Ankerst D. P. (2016). Finasteride reduces risk of bladder cancer in a large prospective screening study. Eur. Urol. 69, 407–410. 10.1016/j.eururo.2015.08.029 26320383

[B30] Mossanen M. Gore J. L. (2014). The burden of bladder cancer care: direct and indirect costs. Curr. Opin. Urol. 24, 487–491. 10.1097/MOU.0000000000000078 24887047

[B31] Nardelli C. Aveta A. Pandolfo S. D. Tripodi L. Russo F. Imbimbo C. (2024). Microbiome profiling in bladder cancer patients using the first-morning urine sample. Eur. Urol. Open Sci. 59, 18–26. 10.1016/j.euros.2023.11.003 38298766 PMC10829607

[B32] Russo F. Esposito S. Tripodi L. Pandolfo S. D. Aveta A. Amato F. (2024). Insights into Porphyromonas somerae in bladder cancer patients: urinary detection by ddPCR. Microorganisms 12, 2049. 10.3390/microorganisms12102049 39458358 PMC11509927

[B33] Sathianathen N. J. Fan Y. Jarosek S. L. Lawrentschuk N. L. Konety B. R. (2018). Finasteride does not prevent bladder cancer: a secondary analysis of the Medical Therapy for Prostatic Symptoms Study. Urol. Oncol. 36, 338.e13–338.e17. 10.1016/j.urolonc.2018.03.020 29731413

[B34] Shen J. Chen C. Chen Z. Gong P. Lee L. S. Schmeusser B. N. (2023). CCL5 promotes the proliferation and metastasis of bladder cancer via the JAK2/STAT3 signaling pathway. Transl. Androl. Urology 12, 1845–1858. 10.21037/tau-23-540 PMC10772649 38196701

[B35] Shibata Y. Arai S. Miyazawa Y. Shuto T. Nomura M. Sekine Y. (2017). Effects of steroidal antiandrogen or 5-alpha-reductase inhibitor on prostate tissue hormone content. Prostate 77, 672–680. 10.1002/pros.23315 28145028

[B36] Tripathi A. Gupta S. (2020). Androgen receptor in bladder cancer: a promising therapeutic target. Asian J. Urol. 7, 284–290. 10.1016/j.ajur.2020.05.011 32742928 PMC7385521

[B37] Wang C.-S. Li C. C. Juan Y. S. Wu W. J. Lee H. Y. (2020). 5α-reductase inhibitors impact prognosis of urothelial carcinoma. Bladder Cancer 20, 872. 10.1186/s12885-020-07373-4 PMC7488389 32917158

[B38] Wang K. Jin S. Fan D. Wang M. Xing N. Niu Y. (2017). Anti-proliferative activities of finasteride in benign prostate epithelial cells require stromal fibroblasts and c-Jun gene. PLoS ONE 12, e0172233. 10.1371/journal.pone.0172233 28196103 PMC5308847

[B39] Wissing M. D. O’Flaherty A. Dragomir A. Tanguay S. Kassouf W. Aprikian A. G. (2021). The use of 5-alpha reductase inhibitors and alpha-1 blockers does not improve clinical outcome in male patients undergoing radical cystectomy for bladder cancer in quebec, Canada. Clin. Genitourin. Cancer 19, 371–371.e9. 10.1016/j.clgc.2021.01.007 33676834

[B40] Wissing M. D. Santos F. Zakaria A. S. O’Flaherty A. Tanguay S. Kassouf W. (2019). Short- and long-term survival has improved after radical cystectomy for bladder cancer in Québec during the years 2000-2015. J. Surg. Oncol. 119, 1135–1144. 10.1002/jso.25456 30919984

[B41] Wissmiller K. Bilekova S. Franko A. Lutz S. Z. Katsburg M. Gulde S. (2023). Inceptor correlates with markers of prostate cancer progression and modulates insulin/IGF1 signaling and cancer cell migration. Mol. Metab. 71, 101706. 10.1016/j.molmet.2023.101706 36931467 PMC10074927

[B42] Xiang P. Du Z. Hao Y. Guan D. Liu D. Yan W. (2021). Impact of androgen suppression therapy on the risk and prognosis of bladder cancer: a systematic review and meta-analysis. Front. Oncol. 11, 784627. 10.3389/fonc.2021.784627 34970495 PMC8712679

[B43] Zhu D. Srivastava A. Agalliu I. Fram E. Kovac E. Z. Aboumohamed A. (2021). Finasteride use and risk of bladder cancer in a multiethnic population. J. Urol. 206, 15–21. 10.1097/JU.0000000000001694 33617325

